# Editorial: Case-reports, a compendium of useful ideas for our daily activity

**DOI:** 10.3389/fsurg.2022.1026401

**Published:** 2022-10-04

**Authors:** Nuria Maria Novoa

**Affiliations:** Thoracic Surgery Department, University Hospital of Salamanca, Institute of Biomedical Research of Salamanca (IBSAL), Salamanca, Spain

**Keywords:** case-report, clinical cases, scientific communication, medical literature, peer-review activity

**Editorial on the Research Topic** Case-reports, a compendium of useful ideas for our daily activity

Back in the first half of the 20th century and before, case-report and case-report series comprised the most relevant type of manuscripts of the medical literature (1). No doubt that our current medical literature is based on robust scientific methodology making case-reports an outlier to it. However, case-reports fill in a broad area of knowledge difficult to complete with other types of publications. A good clinical case-report focus on an exceptional situation and discuss it with special depth adding a good literature review of the topic. But above all, they present an unusual pathology or circumstance for which no clear or determinant data exists, sometimes raising new hypothesis for research and always full of educational value. They transform an anecdote into evidence. These are important reasons to keep case-report and case-report series as a relevant part of our medical reading (2).

Case-reports are a literature genre and as such, writing a good report has its methodology (3). Authors should always keep in mind the main message posed by the clinical case, why is it noteworthy. This type of paper should be short in length to really focus on the problem including only relevant data and avoiding unnecessary or confusing details. Despite the summary effort, this type of paper is easier to write than most of our current scientific manuscripts. They offer a good opportunity to junior clinicians or researchers to initiate a scientific career.

Peer-reviewed manuscripts offer the warranty of a high-quality publication that adds knowledge to our background. It is true that no causal inference or generalization normally is possible (2). However, it raises doubts and/or new ideas basic for future advances and it offers solutions to difficult problems when dealing with rare disorders, for instance. Although, bias is a great problem as journals tend to publish positive-outcome findings (2), some authors have started performing combined analysis showing the value of the case-reports compared to clinical studies in complex meta-analysis (4). This new tool opens the door to increase the current value of this type of publication.

In conclusion, I would say that clinical case-reports are still relevant for the medical community. Although, at a certain moment, it was thought they were going to disappear, we are clearly aware they cover a broad area of knowledge not covered by anything else and due to their clear educational value, among others, clinical case-reports will remain an important tool to improvement. Following these ideas, you will find a nice collection of case-reports included in this section that offer new insights to a variety of thoracic surgery problems. From very unusual cases such as the primary pleural squamous cell carcinoma or a bilateral diaphragm rupture to a easy close situation such as the splenic rupture during a VATS lung resection. You will find a group of interesting and well-sorted out clinical cases. I hope they are to your liking and learn from them.
